# Annealing Foil

**Published:** 1857-06

**Authors:** F. A. Brewer

**Affiliations:** Fulton, Mo.


### ANNEALING FOIL.

BY DR. F. A. BREWER, OF FULTON, MO.

Messrs. Editors:—Allow me to write a few lines, and although they may not be of any importance, yet there may be some who have not adopted the proper mode of annealing gold foil, but have done it carelessly, and pronounced it unworthy of their attention. I have found by experience that foil, or annealed foil, may be used with more advantage in most of cases, I will dare to say, than any other material in use. It has been discussed in our dental meetings, as you are aware, some speaking in favor of it, and these are but few, and the reason for that is because it has not been fairly tested. I can send a filling right home, as it were. I find, or think, it adheres to the bone with more tenacity than the usual foil. I have had thus far no trouble with my annealed foil adhering to the instrument—that being a complaint with some others. My mode of annealing is this: I use a sheet iron box, two inches in length by one inch in width, yet others can have them of the size suitable to their notion. My box has a cover which has four or five small holes in it, to allow the escape of the vapor, if there may be any. The box would be better if it were the length of the leaf of foil, so that in ribbons it will lie in without any binding. When cylinders are used, I make them of different sizes, but all pretty small, as they will give way better to the instrument, and can be packed in the indentations of the bone better than if otherwise. I have read, in the journals, of Dentists making the cylinder of the size of the cavity. In that case, I can not see how it can be packed in the corners, especially in an irregular cavity. I think that foil, annealed in the box, with cover, heated to a red heat, can be used with greater advantage than in any other way. I have tried laying the foil on the sheet of iron, and heating it, but do not think it so good as in the other manner. I have put my box in the stove to heat it, as in cool weather it has been the most convenient. Some practitioners may not give this mode of treatment a fair test, from the little more time and trouble it may cost them ; but I think it pays for itself in the packing. It does not clog and become hard whenever it may be touched with the instrument while packing; but by pressing hard enough, it will become hard enough. I also do not think it scales off in finishing, as other foils. I have also found by experience that the less the foil is touched with the bare fingers, the better it is in every respect, and favor Dr. Forbes's idea in regard to silk gloves.

